# Biliopancreatic Endoscopy in Altered Anatomy

**DOI:** 10.3390/medicina57101014

**Published:** 2021-09-25

**Authors:** Ilaria Tarantino, Giacomo Emanuele Maria Rizzo

**Affiliations:** 1 Endoscopy Service, Department of Diagnostic and Therapeutic Services, IRCCS-ISMETT, 90127 Palermo, Italy; giacomoemanuelemaria.rizzo@community.unipa.it; 2 Section of Gastroenterology & Hepatology, Department of Health Promotion Sciences Maternal and Infant Care, Internal Medicine and Medical Specialties, PROMISE, University of Palermo, 90133 Palermo, Italy

**Keywords:** US, interventional EUS, ERCP, biliopancreatic endoscopy, CH-EUS, FNB, difficult biliary stones

## Abstract

*Background and Objectives*: Anatomical post-surgical alterations of the upper gastrointestinal (GI) tract have always been challenging for performing diagnostic and therapeutic endoscopy, especially when biliopancreatic diseases are involved. Esophagectomy, gastrectomy with various reconstructions and pancreaticoduodenectomy are among the most common surgeries causing upper GI tract alterations. Technological improvements and new methods have increased the endoscopic success rate in these patients, and the literature has been rapidly increasing over the past few years. The aim of this systematic review is to identify evidence on the available biliopancreatic endoscopic techniques performed in the altered post-surgical anatomy of upper GI tract. *Materials and Methods*: We performed a systematic search of PubMed, MEDLINE, Cochrane Library, and SCOPUS databases. Study-level variables extracted were the last name of the first author, publication year, study design, number of patients, type of post-surgical anatomical alteration, endoscopic technique, success rate and endoscopic-related adverse events. *Results*: Our primary search identified 221 titles, which was expanded with studies after the citation search. The final full-text review process identified 52 articles (31 retrospective studies, 8 prospective studies and 13 case reports). We found several different techniques developed over the years for biliopancreatic diseases in altered anatomy, in order to perform both endoscopic ultrasound (EUS) and endoscopic retrograde cholangiopancreatography (ERCP). They included enteroscopy-assisted ERCP (double and single balloon enteroscopy-ERCP, spiral enteroscopy-ERCP) laparoscopic assisted ERCP, EUS-Directed transgastric ERCP, EUS-directed transgastric intervention, gastric access temporary for endoscopy, and percutaneous assisted trans prosthetic endoscopic therapy. The success rate was high (most of the techniques showed a success rate over 90%) and a low rate of adverse events were reported. *Conclusions*: We suggest the considerationof the novel techniques when approaching patients with altered anatomy who require biliopancreatic endoscopy, focusing on the surgery type, success rate and adverse events reported in the literature.

## 1. Introduction

Anatomical gastrointestinal (GI) alterations have always been challenging for performing endoscopy, both in case of diagnostic and therapeutic procedures. Moreover, surgery of the upper GI tract is indicated in different conditions, from oncologic to metabolic and bariatric ones. While the aim of the oncologic surgery is the radical tumor resection, a proper modelling of the stomach and adequate anatomical reconstruction of small bowel are the key to bariatric surgery, with the goal to reduce cost and encourage metabolic changes. Overall, esophagectomy, gastrectomy (with its variants) and pancreaticoduodenectomy are among the most common surgeries causing upper GI tract alteration. Many GI tumors among the approximately 22,000 gastric cancers, 60,000 pancreatic cancers and 19,000 esophageal cancers diagnosed annually in the United States [1,2] require demolitive surgery. In addition, obesity has presently emerged as a western pandemic, so much that bariatric surgery for severe obesity or other metabolic diseases is among the most commonly performed GI interventions. Over the past few years, technological improvements and new methods have increased the endoscopic success in those patients with altered anatomy. Surely, a proper knowledge of the anatomical alterations has been fundamental to perform endoscopy in those patients. In 2011 the global total number of bariatric surgeries was approximately 340,000 [3], and among them Roux-en-Y-Gastric Bypass (RYGB) exceeded other bariatric procedures by 70–80% [4]. In addition, about one-third of post bariatric patients develops gallstones [5]. Furthermore, patients with altered anatomy may also develop those biliopancreatic disorders, which require advanced endoscopy, as endoscopic ultrasound (EUS) or endoscopic retrograde cholangiopancreatography (ERCP). On one hand, a GI post-surgical alteration anatomy may represent for EUS an unpassable hurdle for pancreatic examination and tissue acquisition (TA), because of the difficulty in achieving adequate scans of the pancreas or the distal bile duct, while on the other, it could be insurmountable to achieve the papillary region or the bilioenteric anastomosis during standard ERCP. The aim of this systematic review is to identify evidence on the available biliopancreatic endoscopic techniques performed in the altered post-surgical anatomy of the upper GI tract.

## 2. Materials and Methods

### 2.1. Search Strategy

This systematic review was performed in accordance with the preferred reporting items for systematic reviews and meta-analyses (PRISMA) statements [6]. A systematic search of PubMed, MEDLINE, Cochrane Library, and SCOPUS databases was performed using the following string: “endoscopy” and “altered anatomy” and (“ERCP” or “ultrasound” or “EUS” or “drainage”). The search included reports published from 1 January 2000 to 31 July 2021.

### 2.2. Study Selection

Considering the rareness and new insights of this field in the literature, we included both prospective and retrospective cohort studies as well as randomized controlled trials, and case series or reports. We considered studies to be eligible for this review if they met the following criteria: English language; full-text publications; clear explanation of the altered anatomy of the upper GI tract; presence of success rate as outcome and understandable explanation of the applied endoscopic technique. We excluded studies published in abstract form only, if the reported data were insufficient for an appropriate description of endoscopic technique and patients’ outcomes, and for a full assessment of clinical and technical success. Moreover, we also excluded review articles, editorials, letters to the editor and animal studies. To identify additional studies, the computer search was supplemented with manual searches of the reference lists of all reviewed articles and primary studies retrieved. Duplicate records were removed.

### 2.3. Data Extraction

Study-level variables included the last name of the first author, publication year, study design, number of patients, type of post-surgical altered upper gastrointestinal anatomy, endoscopic technique, success rate and endoscopic-related adverse events (E-AEs). We did not look for assessment of study quality because of the aim of this review was to show all the available data in literature about this overspecialized topic. Success rate was differently defined among studies and depended on the endoscopic procedures performed (Table S1, Supplementary Materials).

## 3. Results

### 3.1. Literature Search

**Figure 1:**
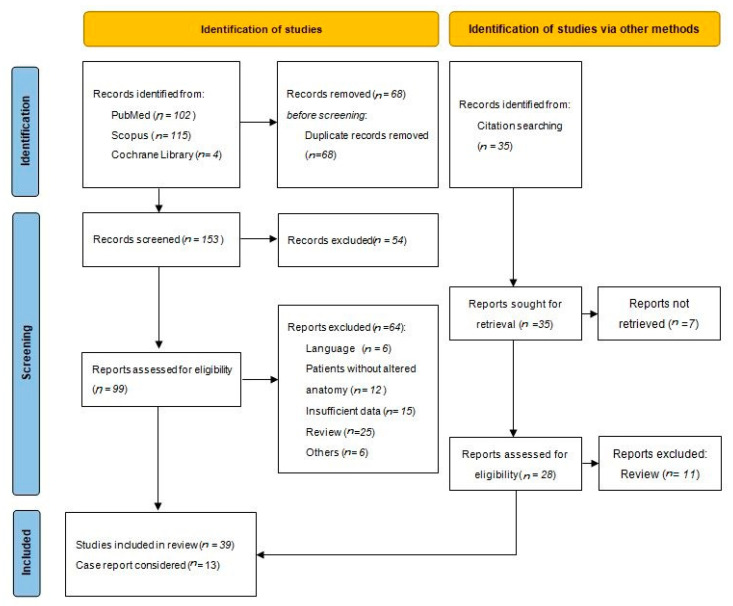
Flowchart Systematic Review.

Our primary search identified 221 titles. After removal of duplicate articles, we identified 153 studies. We excluded 54 articles because not pertinent. Finally, 99 studies were included in a qualitative synthesis and full-text review process. Another 17 studies were added with citation search, after a similar screening and review process (two oral abstract were exceptionally included for their completeness of data). After complete review of the studies, 52 articles (31 retrospective studies, 8 prospective studies and 13 case reports) fulfilled the inclusion criteria (Figure 1). No randomized controlled trials were found.

Our review process showed bias in the selected studies regarding patient selection and technical and clinical success rate definition, as deeply discussed in each following section. There was complete concordance between reviewers for study selection and data abstraction.

### 3.2. Surgical Anatomical Variant

Indications for upper GI surgery may include both malignant and benign indications, as peptic ulcer or dysmetabolic diseases in the latter case. Surgical techniques causing a higher difficulty in the post-surgery endoscopic management mainly involve the gastro-duodenal portion, even if esophageal surgery may determine relevant alteration of the anatomy as well. Total or distal esophagectomy, total or sleeve gastrectomy, partial gastrectomy with different reconstruction procedures (i.e.,Billroth I, Billroth II, Roux-en-Y reconstruction) and pancreaticoduodenectomy with its variants are the most involved in relevant anatomic alteration for endoscopic procedures. Techniques and altered anatomy after-surgery are defined in Table S2.

### 3.3. Diagnostic Endoscopic Ultrasound in Altered Anatomy

Endoscopic ultrasound (EUS) is nowadays routinely performed as valuable procedure for detection, staging, and cytohistological characterization of biliopancreatic diseases. An altered anatomy of upper GI tract may represent an issue for an appropriate pancreatic examination and tissue acquisition (TA), due to the difficulty in achieving a proper scan of the pancreas or the bile duct. The quality of endosonographic image resolution is dependent on the proximity of the transducer to the biliopancreatic region, so successful EUS in altered anatomy depends on the knowledge of the anatomic post-surgery alteration and endosonographer experience. Moreover, even experienced endosonographers may not be able to find the way to obtain adequate window, and to move an echoendoscope through an altered anatomy, especially when anastomotic reconstructions are unclear or particularly laborious. Combined techniques consider other access to the biliopancreatic region, in order to avoid anatomic alterations. In fact, Bowman et al. [7] analyzed patients who required laparoscopic biliopancreatic endoscopy, mainly for choledocholithiasis, presenting five patients who performed diagnostic laparoscopic EUS (LA-EUS) before laparoscopic ERCP (LA-ERCP) with a success rate of 100%. Moreover, diagnostic EUS with TA in altered anatomy could be difficult, but surgical tissue acquisition should be considered after at least an endoscopic attempt due to its invasiveness. A decade ago, Wilson et al. [8] showed a success rate of EUS-TA of 73.94% among 188 patients with heterogeneous surgical alterations (Billroth I, Billroth II, Roux-en-Y, gastric bypass, Whipple, Puestow, Nissen fundoplication, esophagectomy) with no AEs reported. More recently, a retrospective study of 242 patients showed a rate of AEs after diagnostic EUS of 1.24% and an overall technical success rate of 78.2%. Actually, the EUS technical success rate was shown to vary depending on the different surgical alterations.In fact, a low success rate was seen in the Roux-en-Y gastric bypass (62.5%) and total gastrectomy (66.7%), while a high success rate was showed in sleeve gastrectomy and Billroth I anastomosis (100 and 95.7%, respectively). In general, TA-failure may also happen due to various reasons, including failed visualization, lesions too deep to be punctured or lesions being impossible to penetrate.

### 3.4. Biliopancreatic Interventional Endoscopy in Altered Anatomy

#### 3.4.1. Endoscopic Retrograde Cholangiopancreatography (ERCP)

ERCP on surgically altered anatomy is laborious, technically difficult and associated withhigher rate of failure and adverse events in comparison with standard procedures, especially in those with most complex reconstructions [9]. First reports of ERCP techniques in altered anatomy dated back to 40 years ago, with attempts in using a pediatric colonoscope in Roux-en-Y anatomy [10]. Later, Elton et al. described their use of a pediatric colonoscope and enteroscope for diagnostic and therapeutic intervention in long limb bypass patients, with an overall success rate of 84% and cannulation rate of 94%. Despite the high success rates, technical disadvantages included the lack of side viewing orientation and an elevator, and a channel size that precluded the use of conventional stents and accessories [11]. Among the first reports on the use of conventional duodenoscope in altered anatomy, Hintze et al. reported a success rate of only 33% in reaching the papilla in RYGB, and 67% in patients with Billroth II anastomoses. [12] The use of a forward-viewing colonoscope and the duodenoscope in long limb Roux-en-Y gastrojejunostomy patients to perform ERCP was later reported by Wright et al. with a 67% of ERCP success rate [13]. Given the challenge, the development of new tools to improve procedural success remainsthe goal. In fact, instrumental upgrades haves been attempted over the years: multibending backward–oblique-viewing duodenoscope, [14] variable-stiffness duodenoscope, [15] and multibending forward-viewing endoscope (M-scope) [16], but have yet to become routinely used in clinical practice. Alternative techniques consider single-balloon and double-balloon enteroscopes to perform ERCP in altered anatomy, but the long endoscope length limits the use of conventional ERCP accessories [17]. For this reason, the short-type single-balloon and double-balloon enteroscopes have been developed as alternatives [18]. Even if the underwater technique is primarily used to perform colonoscopy, the underwater-ERCP using a cap-assisted pediatric colonoscope was recently proposed in six patients with altered anatomy as an alternative, achieving a success rate of 100% without any AEs [19]. Furthermore, many case reports have been published during the last decade, showing alternative techniques for ERCP in different scenarios, varying from management of Mirizzi syndrome in Billroth II reconstruction to cholangiocarcinoma in RYGB [20,21,22,23,24,25,26,27]. In 2006, the short length double-balloon enteroscopy (s-DBE) was firstly used to perform ERCP in RYGB patients [28]. Since then, the technique has been based on using the short type of endoscope in order to permit to use ERCP accessories (155 cm of length, with a working channel of 3.2 mm). Later, in 2008, ERCP in RYGB patients was reported with the single balloon (SBE) tip overtube, which had a length similar to the long DBE scope (200 cm) with a thinner working channel (2.8 mm). These techniques have showed different success rates over the years, depending mostly on the surgical alterations. Overall, sDBE-ERCP had a success rate between 70.7 and 96% [29,30,31,32,33,34,35], while SBE-ERCP appeared to be as effective as sDBE, with a success rate among 73 and 92.3% [36,37,38,39]. Among studies about SBE-ERCP, Lenze et al. [40] in a prospective single-center study, showed a lower success rate (57.7%), but they also found in univariate analysis that malignant biliary obstruction had a significantly higher risk of SBE-ERCP failure (OR = 11.33, *p* = 0.001). Another alternative enteroscopy-assisted technique is spiral enteroscopy (SE), which Ali [41] and Zouhairi [42] demonstrated to be successfull among RYGB patients to reach the papilla in 86% and 76.2% of cases, with an overall success rate for SE-ERCP of 86% and 64.3%, respectively. Moreover, a retrospective study compared SBE-ERCP and SE-ERCP on 54 patients with Roux-en-Y anatomy, showing similar diagnostic and therapeutic yield (diagnostic yield of 48.3% and 40%, respectively), and no significant differences on the rate of E-AEs (only one AE after SBE-ERCP) [43]. Table 1 summarizes these data. In addition, a metanalysis of 1523 patients in 2015 showed a pooled procedural success rate of 93% (95% CI 88–97%), and a subgroup analysis (short DBE and long-scope DBE) with a procedural success rate of 96% (95% CI 91–100%) and 88% (95% CI 76–96%), respectively [44]. In conclusion, ERCP in altered anatomical condition showed a technical improvement over the years using different scopes. Moreover, it was expected to reach a more satisfied result, but unfortunately it is still irregular among studies, likely depending on the following factors: lack of standardized technique, lack of predictors of success and lack of correlation between anatomical alteration and specific ERCP instrument (single/double balloon enteroscope, spiral enteroscope, duodenoscope). Furthermore, every anatomical alteration could be different and not completely predictable. Despite the difficulty of the techniques, AEs seem to be acceptable, with the highest rate when sDBE-ERCP is performed (17.6% the worst rate in the studies evaluated).

#### 3.4.2. EUS-Guided Procedures

Although different aforementioned techniques have been proposed over the years to perform biliopancreatic endoscopy in altered anatomy, improving technical success still needs some implementation and alternatives. Therefore, EUS-guided or assisted procedures to perform ERCP are increasing and many case reports without routine solutions have been reported over the years [55,56,57,58,59], both to get access to the biliary limb and for directly performing the procedure. Recently, a water-filled diagnostic and therapeutic EUS procedure has been proposed for patients with Billroth II or Roux-en-Y reconstruction, in order to achieve a higher success rate and lower adverse event rate, but few cases are described in literature to properly understand its efficacy [60]. In the last decade, a novel technique has been developed in RYGB patients, the EUS-directed transgastric ERCP (EDGE). Kedia et al. [61] proposed the initial technique as a two-stage procedure (double stage EDGE): firstly, inserting a percutaneous gastrostomy (PEG) tube in the excluded stomach after the EUS-assisted identification and distension of the excluded cavity through the pouch; later, the PEG-tube was exchanged for a fully covered self-expanded metal stent (FCSEMS) and anterograde ERCP was performed via the percutaneous FCSEMS. This technique did not propagate as expected due to some limitation, as the risk of PEG site infection (two of the six patients reported in Kedia’s series experienced PEG site infection) and the inability to perform it in case of urgency (i.e., cholangitis). A year later, Kedia et al. [62] improved their technique with the development of the single-stage EDGE (SS-EDGE) thanks to the spread of the Luminal Apposing Metal Stent (LAMS) in clinical practice. In fact, the upgraded technique entails the creation of a gastro–gastric (G–G) or jejunogastric (J-G) fistula with the excluded stomach through a EUS-guided LAMS placement, avoiding the percutaneous access. This case series of five patients with RYGB treated with SS-EDGE reported a technical success of 100% using the 15 mm diameter LAMS, even if initially two of five patients showed difficulty in passing the duodenoscope through the LAMS and three of the five experienced stent migration. Nonetheless, severe AE were not reported. The technique contemplates the use of either over-the-wire (OTW) LAMS placement or the freehand technique to release LAMS, depending on expertise and availability of the centers. The studies about EDGE reported an extremely high success rate of this novel technique, between 96.5 and 100% [63,64,65,66,67,68,69,70]. Adverse events include mostly LAMS maldeployment and migration, which seemed to be mainly seen in those studies in which authors used the OTW technique. The freehand technique seems to give an advantage in terms of LAMS migration. Furthermore, EDGE and enteroscopy assisted ERCP (E-ERCP) were compared in a multicenter study, which reported a technical success higher with EDGE when compared to E-ERCP (100%vs. 60%, *p*< 0.001), with relatively similar rate of E-AEs (6.7% vs. 10.0%, *p* = 1) [66]. However, EDGE created an alternative to laparoscope assisted ERCP (LA-ERCP) so comparative data have been reported over the years. In 2018, a meta-analysis comparing LA-ERCP and EDGE including 941 patients (843 LA-ERCP and 98 EDGE) showed pooled technical and clinical success rates similar in both of the groups (98% vs. 96%, *p* = 0.07 and 96% vs. 96%, *p* = 0.84). AE rate had no significant difference (13% vs. 10%, *p* = 0.32) [71]. Data reported by Khara et al. confirmed a high success rate among 76 patients in both those who performed LA-ERCP and EDGE (both 100%), with no significant difference in adverse events rate (17%vs. 6%, *p* = 0.94), even if LA-ERCP had a slightly higher percentage of AEs [72]. However, variants of EDGE technique have been recently proposed.In 2019, Wang et al. reported a case series (10 patients) in which LAMS was exchanged with double pigtail plastic stents at the end of the procedure or after a follow up period in order to permit closure of the fistula. The authors defined the “Gastric Access Temporary for Endoscopy” approach (GATE) [73]. In the same year, Krafft et al. proposed to take advantage of the transgastric (anterograde) approach of EDGE in order to extend the indications to other biliopancreatic and luminal disease through EUS-directed interventions, naming it as “EUS-directed transgastric intervention” (EDGI). Among 14 patients, a diagnostic EUS of extraluminal pathology was indicated in 42.7% and endoscopic biopsy of gastroduodenal luminal abnormalities in 35.7%. In those cases in which the freehand LAMS deployment technique was applied (71.4%), no LAMS maldeployment was seen, while two cases occurred when OTW technique were performed [74]. A rare-reported AE of EDGI was the dehiscence of the anastomosis, even if it occurred after polypectomy performed near the pylorus through the jejuno-gastric anastomosis [75]. These data are summarized in Table 2.

#### 3.4.3. Alternative Access

Alternative techniques for permitting biliopancreatic endoscopy in altered anatomy have been proposed, including LA-ERCP, percutaneous assisted trans prosthetic endoscopic therapy (PATENT), and the abovementioned EDGE procedure. These alternative techniques permit the use of a conventional duodenoscope with its available standard ERCP accessories. PATENT permits to achieve direct access to the GI area were excluded after surgery, as the biliopancreatic area, and can be performed with device-assisted enteroscopy (DAE) or EUS-guided endoscopy. More precisely, PATENT technique entails the creation of a percutaneous access to GI tract in order to facilitate reaching the area of interest. DAE-PATENT consists of deploying a percutaneous gastrostomy (PEG) tube in the excluded stomach of RYGB patients through an enteroscope, with subsequent performance of ERCP via the PEG. Data from a retrospective case series of five patients showed technical success in all of the procedures attempted and only one AE [54]. The other way to perform PATENT is the EUS-guided technique, but few data are available in the literature about this EUS-guided gastrostomy application and they are mainly pilot studies [76,77]. The EUS-PATENT technique consists of the ultrasound identification of the remnant stomach through the gastric pouch (ultrasound visualization of the sand dollar sign helps to correctly identify the excluded stomach [78]), EUS-guided puncture of remnant cavity and filling it with contrast and carbon dioxide. These maneuvers are necessary to percutaneously detect the excluded stomach in order to correctly insert the devices for PEG insertion. These techniques are obviously not routinely used in clinical practice, because they need a tertiary center and endoscopists with high expertise. Another way to get direct access to the excluded GI area with the scope is performing LA-ERCP, which was first described about 20 years-ago by Peters et al. [79]. This procedure entails a laparoscope-assisted surgical port placement into the excluded stomach, followed by percutaneous passage of the duodenoscope via the lap port into the duodenum. Grimes et al. [47] reported a success rate of 95% of LA-ERCP performed in 38 patients with RYGB, with 13% of AEs. Later, Bowman et al. reported data about 16 patients with RYGB, 11 of whose experienced LA-ERCP and the other five experienced the combined LA-EUS plus LA-ERCP. Success rate was 100% with 0% of AEs, confirming not only the efficacy of LA-ERCP but also the availability of LA-EUS as a diagnostic tool [7]. Moreover, in a series of eight patients with RYGB, intragastric single port surgery (IGS) was reported to be effective and safe for LA-ERCP (100% success rate and 0% of E-AEs) [52]. In general, a systematic review from 26 studies regarding trans gastric ERCP in patients with RYGB reported 100% success gastric access and 98.5% success ductal cannulation, but the access to the excluded stomach was achieved differently (laparoscopically in 58% of reported cases, open surgery in 6%, by antecedent gastrostomy tube placement in 33%, and with EUS-assistance in the 3% of residual cases) [80].

## 4. Discussion

In biliopancreatic endoscopy, the difficulty of the procedure is further increased in the presence of surgically altered upper GI anatomy and it becomes a challenge for endoscopists. The literature regarding this topic has been increasing over recent years, especially during the last decade. Probably, this phenomenon may be explained in part by a high incidence and prevalence of obesity with the consequent increase of bariatric surgeries. Furthermore, the increase of expertise and the improvement of endoscopic devices and techniques may be more encouraging for physicians to present their data and innovations than have been previously. Another aspect regards the surprising high success rate of the novel procedures, especially if we consider that biliopancreatic techniques are generally complex in a normal anatomy. Many explanations may be given: endoscopists who perform these new techniques are high-level specialists, a staff highly skilled is implicated in the management of these patients at tertiary centers, or perhaps because the success rate is differently defined, providing high heterogeneity among studies. In fact, a standardized definition of technical and clinical success rates is lacking because it varies through the studies or iseven specified in some of them. Furthermore, this review has some limitations: firstly, it consists of data from different types of studies, and thus was useless for appraising the quality of individual studies or for searching indicators of heterogeneity. Our choice to avoid a more restricted selection criteria was based on the awareness that this is an overspecialized field, so indication and technique are not standardized and guidelines lack recommendations. As a result, including most of the data available in literature would have given an advantage for a more complete and realistic review. Another limitation is that many data come from small cohorts, while studies with large data as multicenter are still few. Another bias regards patient selection, because there are several different post-surgery anatomical alterations providing heterogeneity, although patients with Roux-en-Y reconstruction are included in several studies.

## 5. Conclusions

In conclusion, recent evidence suggests the consideration of the novel techniques currently available when approaching patients with altered anatomy who require biliopancreatic endoscopy. The choice of the technique should take into consideration local expertise, previous surgical intervention, indication and the reported success rate in literature. Moreover, a multidisciplinary approach should be routinely applied, with the collaboration among gastroenterologists, radiologists and surgeons in order to better manage the most complex ones among those biliopancreatic patients with altered anatomy. Finally, standardization of outcomes, in terms of technical and clinical success, is mandatory to make results comparable and applicable to clinical practice.

## Figures and Tables

**Table 1:** Success rate of different therapeutic endoscopic biliopancreatic techniques in altered anatomy.

First Name, Year	Article Type	N. of Patient	Type of Surgery	Indication	Endoscopic Technique	Technical Success	E-AEs Reported
Brozzi [45], 2021	Retrospective study	242	86 (35.5%) Billroth II; 77 (31.8%) PD; 23 (9.5%) Billroth I; 19 (7.9%) distal esophagectomy; 15 (6.2%) total gastrectomy; 14 (5.8%) sleeve gastrectomy; 8 (3.3%) Roux-en-Y.	Suspected solid pancreatic lesions documented on cross-sectional imaging (46.7%), suspected cystic pancreatic lesions (18.2%), suspected choledocholithiasis (10.3%), post-pancreatic resection follow-up (6.2%), unexplained CBD dilation in 14 (5.8%), main pancreatic duct dilation (4.9%), suspected extrahepaticcholangiocarcinoma (2.1%), chronic pancreatitis (2.1%), pancreatic cancer screening (2.1%), idiopathic recurrent pancreatitis (1.6%).	Conventional EUS	Overall: 78.2% **TA–success rate:** 71.3%	3 (1.24%)
Ishihara [29], 2021	Retrospective study	73	PD with pancreaticojejunostomy and HJ: bowel reconstruction methods were Child in 46 (63%), Roux-en-Y in 22 (30%),and other in 5 (7%).	Intrahepatic bile duct stones	sDBE-ERCP	92%	6.8%
Kogure [30], 2021	Retrospective study	40	Surgical reconstructions: 26 Billroth-II, 13 Roux-en-Y, and 1 Imanaga	18 pancreatojejunostomy anastomotic stricture (PJAS), four pancreatic duct stone (PDS), 4 pancreatic fistula (PF), 11 PJAS with PDS, 3 PJAS with PF.	sDBE-ERCP and EUS-PD	sDBE-ERCP 70.7% (29/41) EUS-PD: 100% (9/9)	12.2%
Sato [31], 2020	Retrospective study	102	Roux-en-Y 80 (78.4%), Billroth-II 22 (21.6%)	HJ anastomotic strictures	sDBE-ERCP	89.2%	17.6%
Mukai [46], 2019	Retrospective study	48	9 gastrectomy with Roux-en-Y. 2 gastrectomy with Billroth-II. 17 HJ with Roux-en-Y. 3 hepaticoduodenostomy. 6 PD with Whipple	Benign biliary diseases: common bile duct stones [*n* = 11], intrahepatic bile duct stones [*n* = 5], anastomotic strictures [*n* = 21]	EUS-guided antegrade intervention	91.9%	8.1%
Fujimoto [32], 2018	Retrospective study	102	Gastrectomy + R-Y (38/102); Gastrectomy + B-II (24/102); PD (23/102); HJ+R-Y (17/102)	CBD stones, anastomotic stricture of HJ, IHBD stones, chronic pancreatitis and pancreatic stone, cholangitis, stenosis of afferent loop	sDBE-ERCP or a regular gastroendoscope	80%	1.96%
Yamada [33], 2019	Prospective collected data–Propensity score matched patients	326	Gastrectomy with B-II, gastrectomy with R-Y, PD with B-II, PD with R-Y, HJ with R-Y, and liver transplantation with HJ	Biliary strictures, anastomosis stenoses, choledocholithiasis, intrahepatic stones, obstructive jaundice, bile duct leaks, pancreatic duct leaks, chronic pancreatitis with pancreatic duct strictures, intraductal pancreatic stones	sDBE-ERCP and cDBE-ERCP	Short-type DBE: 150 (92%) cDBE: 145 (89%)	5.52%
Bowman [7], 2016	Retrospective study	16	RYGB	Choledocholithiasis, CBD stenosis, recurrent acute pancreatitis, stone IOC, gallstone pancreatitis	LA-ERCP: 11 cases Combined LA-EUS plus LA-ERCP: 5	100%	0%
Grimes [47], 2015 *	Retrospective study	38	RYGB	Chronic abdominal pain, including SOD, pancreatic duct stenosis, chronic pancreatitis, choledocolithiasis	LA ERCP	95%	13%
Bove [48], 2015	Retrospective study	713	Gastrectomy with B-II reconstruction	Common bile duct stones (51.2%) and obstructive jaundice (24.8%)	c-ERCP or gastroscope	93.8%	4.3%
Shimatani [36], 2014	Prospective study	26	4 RYGB,8 R-Y HJ, 3 BII, 3 PD, 6 ppPD, 2 other reconstructions.	NA	sSBE-ERCP	84.6%	3.8%
Tomizawa [37], 2014	Retrospective study	14	Roux-en-Y reconstruction after Whipple procedure (*n* = 4), HJ (*n* = 9) and partial gastrectomy (*n* =1).	Obstructive jaundice (*n* = 10), cholangitis (*n* = 7), post-PTC internalization (*n* = 3) and biliary stent extraction/exchange(*n* =2)	SBE-ERCP	73%	0%
Lenze [40], 2014	Prospective study	26	9 Billroth II with Roux-en-Y; 9 biliodigestive anastomosis with Roux-en-Y; 5 total gastrectomy with Roux-en-Y; 2 pp-Whipple PD; 1 Whipple PD	Obstructive cholestasis: 15 choledocolithiasis: 10 obstruction of pancreatic duct: 1	SBE-ERCP	57.7%	NA
Iwashita [49], 2013	Retrospective Study	7	Total gastrectomy: 3 Subtotal gastrectomy: 2 Pancreaticoduodenectomy: 2	5 Choledocholithiasis,1 malignant biliary obstruction,1 bilioenteric anastomosis stricture	EUS-guided antegrade treatments	100%	28% (2/7)
Lee [50], 2012	Retrospective study	13	Billroth II gastrectomy	Choledocolithiasis	EPBD-ERCP with forward-viewing endoscope	92.3%	0%
Cho [34], 2011	Retrospective study	20	6 patients Billroth II, 7 Roux-en-Ywith HJ, 5 Roux-en-Y with GJ, 1Roux-en-Y with EJ, 1 Whipple’s operation with choledochojejunostomy	Choledocholithiasis, stricture, cholangitis, bile leakage	sDBE-ERCP	24/25 (96%)	NA
Wilson [8], 2010	Retrospective study	188	BI, BII, RYGB, Whipple, Puestow, Nissen fundoplication, esophagectomy	NR	EUS-TA	139/188 (73.94%)	0%
Wang [38], 2010	Retrospective study	13	Whipple (*n* =3), hepaticojejunostomy (*n* =3), Billroth II (*n* =1), and Roux-en-Y (*n* =9)	Cholangitis, choledocholithiasis, biliary pancreatitis, Retained stent from OLT, CBD stricture	SBE-ERCP	92.3%	15.39%
Hakuta [35], 2020	Retrospective study	568	Gastrectomy B-II, Gastrectomy R-Y, PD R-Y, PD B-II, Extrahepatic bile duct resection with R-Y	Bile duct stone, benign biliary stricture, malignant biliary obstruction, cholangitis, pancreatic intervention	sDBE-ERCP	79.93%	10.04%
Fugazza [19], 2020	Prospective study	6	3 (50%) distal Gastrectomy RY, 2(33.3%) with Whipple pylorus preserving and 1(16.7%) with bariatric Gastro-jejunal Bypass	Jaundice or cholangitis secondary to bile duct stones	uERCP°	100%	0%
Yane [39], 2017	Retrospective study	117	BII gastrectomy 13 (11.1), PD 51 (43.6), Roux-en-Y gastrectomy 25 (21.4), HJ with Roux-en-Y 28 (23.9)	Bile duct stone 28 (23.9), bile duct stricture 16 (13.7), stricture of choledo- or hepaticojejunal anastomosis 51 (43.6), stricture of pancreaticojejunal anastomosis 14 (12.0), Others 8 (6.8)	sSBE-ERCP	81.8%	5.9%
James [51], 2018	Retrospective study	20	9 RYGB, 6 Roux-en-Y HJ, 2 Billroth II procedures, and 3 Whipple procedures.	Common bile duct stones (*n* = 8), benign postoperative strictures (*n* = 7), chronic pancreatitis (*n* = 3), inflammatory stricture (*n* = 1), and treatment of a bile leak (*n* = 1)	EUS-guided hepaticoenterostomy	90%	15 5%
Bures [52], 2019	Prospective Study	8	RYGB	Choledocholithiasis	LA-ERCP with intragastric single-port surgery	100%	0%
Ali [41], 2018	Retrospective Study	31	28 in RYGB and 7 “long- limb- RY” surgical reconstructions: 4 in patients with RY-HJ and 3 in patients with gastrectomies and RY reconstructions	Choledocholithiasis 14 (40%); malignant obstruction 6 (17%); SOD 5 (14%); stent placement 2 (6%); Stent extraction 2 (6%); biliary pancreatitis 2 (6%); type III choledochocele 1 (3%); bile leak 1 (3%); HJ stricture 1 (3%); ampullary stricture post prior sphincterotomy 1 (3%)	SE-ERCP	86%	0%
Zouhairi [42], 2015	Retrospective study	42	39 with gastric bypass Roux-en-Y, 2 with Billroth II gastrectomy, and 1 with hepaticojejunostomy associated with liver transplant	Choledocholithiasis: 13 (30.9%), biliary obstruction: 20 (47.6%), suspected sphincter of Oddi dysfunction: 4 (9.5%), abnormal liver enzymes: 1 (2.4%), ascending cholangitis: 2 (4.8%), and bile leak: 2 (4.8%)	SE-ERCP	64.3%	7.69%
Wagh [53], 2012	Prospective study	7	Roux-en-Y HJ 2/7 (29%); RYGB 3/7 (43%); RYGB with HJ 1/7 (14%); BII gastrectomy with Braun enteroenterostomy 1/7 (14%)	Biliary obstruction 5/7 (72%); bile duct stone(s) 1/7 (14%); Pancreatic leak 1/7 (14%)	SE-ERCP	69%	0%
Law [54], 2013	Retrospective study	5	RYGB	SOD (Type I [*n* =3], Type II [*n* =2])	DAE-PATENT	100%	20%

* Only data from initial ERCP laparoscopic assisted were extracted; °The u-ERCP technique consists of the underwater advancement of a pediatric colonoscope with a transparent cap fitted on the tip of the endoscope.NR = Not Reported; PD = Pancreatoduodenectomy; Uercp = Underwater ERCP; CBD = Common Bile Duct; EUS = Endoscopic Ultrasound; HJ = hepaticojejunostomy; sDBE-ERCP = Short double-balloon endoscopy ERCP; EUS-PD = endoscopic ultrasonography-guided pancreatic duct drainage; EUS-PD = endoscopic ultrasonography-guided pancreatic duct drainage; SOD = sphincter of Oddi dysfunction; BII = Billroth II; uERCP = Underwater ERCP; SE-ERCP = Spiral enteroscopy-ERCP; RYGB = Roux-en-Y gastric bypass; GJ = gastrojejunostomy; EJ = esophagojejunostomy; IOC = intra-operative cholangiogram; DAE-PATENT = device-assisted enteroscopy-percutaneous assisted trans prosthetic endoscopic therapy; EPBD = Endoscopic transpapillary large balloon dilation. Bold: includes a subgrooup of result slightly different from “Technical success”.

**Table 2:** Success rate regarding EDGE technique and its variants (GATE and EDGI).

First Name, Year	Article Type	N. of Patient	Type of Surgery	Endoscopic Technique	LAMS Diameter	Technical Success	Clinical Success	E-AEs Reported
Kedia [62], 2015	Prospective study	5	RYGB	SS–EDGE 3 DS-EGDE 2	15 mm	100%	60%	stent dislodgement: 60%
Tyberg [63], 2016	Prospective study	16	RYGB	SS–EDGE 4 DS-EGDE 6	15 mm	100%	91%	Stent migration(19%), 1 jejunal perforation
Ngamruengphong [64], 2017	Retrospective study	13	RYGB	SS–EDGE 2 DS-EGDE 11	15 mm	100%	100%	Stent migration(33%)
James and Baron [65], 2018	Retrospective Study	19	RYGB	SS–EDGE 4 DS-EGDE 15	15 mm	100%	100%	Stent malposition(6/19)
Bukhari [66], 2018	Retrospective Study	30	RYGB	SS–EDGE 8 DS-EGDE 22	15 mm	100%	100%	LAMS migration(6.7%),bleeding(3.3%)
Chiang [67], 2018	Oral Abstract–retrospective study	66	RYGB	SS–EDGE 43 DS-EGDE 23	NR	92.4%	NR	Bleeding (7.6%), LAMS malposition(4.5%), LAMS migration (4.5%), perforation (1.5%), pancreatitis(1.5%)
Kedia [68], 2018	Retrospective study	29	RYGB	NR	15 mm	96.5%	96.5%	Perforation (1),pancreatitis (2)stentdislodgement(3)bleeding (1).
Wang [73], 2019	Retrospective study	10	RYGB	SS–GATE 7 DS-GATE 2	15 mm	100%	100%	Stent migration (20%), bleeding in one patient
Hsueh [69], 2019	Oral Abstract –Retrospective study	9	RYGB	SS–EDGE 2 DS-EGDE 7	20 mm	100%	100%	None
Runge [70], 2020	Retrospective study	178	RYGB	SS –EDGE 85 DS-EGDE 81	NR	98%	NR	Perforation(6), stentmigration(13),bleeding (2), Pneumoperitoneum(3),post ERCP pancreatitis(3), cholangitis (1)
Krafft [74], 2019	Retrospective study	14	RYGB	SS –EDGI 5 DS-EGDI 2	20 mm (*n* =8) 15 mm(*n* =6)	100%	100%	Stentdislodgement(14.3%)
Khara [72], 2021	Retrospective study	76	RYGB	59 LA-ERCP 17 EDGE	20 mm	Both 100%	Both 100%	17% LA-ERCP 6% EDGE

EDGE= EUS-directed transgastric ERCP; E-Aes= Endoscopy-related adverse events. EDGI= EUS-directed transgastric intervention; GATE= Gastric Access temporary for Endoscopy; SS= single stage; DS= double stage; GGF= gastrogastric fistula; LA-ERCP= Laparoscopic–ERCP.

## Data Availability

To be excluded.

## Supplementary Materials

The following are available online at www.mdpi.com/article/10.3390/medicina57101014/s1, Table S1. Different success rate definitions per study, Table S2. Surgical techniques and altered anatomy after-surgery.
