# Supplementary material for: Biliopancreatic Endoscopy in Altered Anatomy
Source: Medicina (Kaunas). 2021 Sep 25;57(10):1014. doi: 10.3390/medicina57101014 (PMC8537222; doi:10.3390/medicina57101014)
Supplement: Supplementary file 1 [file medicina-57-01014-s001.zip › medicina-1372147-supplementary.pdf]

Supplementary Materials

Table S1. Different success rate definitions per study.

First name, year	Procedural/ Technical success	Definition
Brozzi, 2021	Overall: 78.2%	The percentage of cases with satisfactory visualization of the biliopancreatic target lesion/region according to the endosonographer
Ishihara, 2021	92%	Reaching the hepaticojejunostomy site
Kogure, 2021	sDBE-ERCP 70.7% EUS-PD: 100%	DB-ERCP: when scope insertion, pancreatic duct cannulation, and planned interventions were accomplished.<br>EUS-PD: when puncture of the pancreatic duct, insertion of the guidewire, and plastic stent/nasopancreatic catheter placement were achieved
Sato, 2020	89.2 %	The achievement of balloon dilation or plastic stent placement across the stricture via DB-ERCP
Mukai, 2019	91.9 %	The technical success of the creation of a hepatoenteric tract was defined as successful stent placement in the bile duct
Fujimoto, 2018	80 %	The rate of successfully completed ERCP procedures
Yamada, 2019	sDBE: 150 (92%); cDBE: 145 (89%)	Success rate of pancreaticobiliary intervention
Bowman, 2016	100 %	Biliary/pancreatic cannulation and intervention rate
Grimes*, 2015	95 %	Selective cannulation of biliary and/or pancreatic ducts
Bove, 2015	93.8 %	Successful cannulation/opacification of the desired biliopancreatic duct
Shimatani, 2014	84.6 %	The therapeutic success rate was defined as the rate of accomplishment of ERCP-related interventions
Tomizawa, 2014	73 %	Overall SBE-assisted ERCP success rate
Lenze, 2014	57.7 %	Overall success rate
Iwashita, 2013	100 %	Procedure success rate
Lee, 2012	92.3 %	Selective CBD cannulation
Cho, 2011	24/25 (96 %)	Bile duct cannulation
Wilson, 2010	139/188 (73.94%)	Successful imaging
Wang, 2010	92.3 %	The reaching of the ampulla or hepaticojejunostomy
Hakuta, 2020	79.93 %	A successful biliary or pancreatic cannulation, and subsequent stone removal, balloon dilation, or stent placement, if needed
Fugazza, 2020	100 %	Completion of all of the steps of u-ERCP
Yane, 2017	81.8 %	Successful enteroscopy, with successful diagnostic and therapeutic interventions
James, 2018	90 %	Completion of EUS-guided biliary stent placement from left intrahepatic duct into the gastrointestinal tract
Bures, 2019	100 %	Therapeutic success
Ali, 2018	86 %	The success rate for reaching the papilla
Zouhairi, 2015	64.3%	The rate to successful complete the procedure
Wagh, 2012	69 %	Endoscopic therapy successfully performed
Law, 2013	100 %	NR
Kedia, 2015	100 %	Success in completing the procedure
Tyberg, 2016	100 %	Successful deployment of the LAMS
Ngamruengphong, 2017	100 %	Successful placement of the LAMS across the transgastric fistula
James and Baron, 2018	100 %	Success in completing the procedure
Bukhari, 2018	100 %	Successful cannulation of the selected duct with successful intervention as intended
Chian, 2018	92.4 %	NR
Kedia, 2018	96.5 %	The success of excluded stomach access via EDGE gastrogastric fistula creation
Wang, 2019	100 %	Success in completing the procedure
Hsueh, 2019	100 %	Clinical success: successful pancreaticobiliary cannulation and intervention. Technical success: successful fistula creation with 20 mm LAMS.
Runge, 2020	98 %	Successful placement of a transmural LAMS followed by ERCP with cannulation of the intended duct and completion of the intended intervention(s).
Krafft, 2019	100 %	Completion of the diagnostic and/or interventional endoscopic procedure by way of the endoscopically-created transmural fistula
Khara, 2021	100 % (Both LA-ERCP and EDGE)	NR

NR = Not reported

Table S2. Techniques and altered anatomy after-surgery.

Technique	Reconstructive procedures or surgical variants		Surgical technique
Esophagectomy	Total		After esophagus resection, a short segment of the cervical esophagus is sutured with the upper part of the stomach (converted into a long tube), or with the colon or by transplanting a segment of small intestine. There are two approaches: open surgery or mini-invasive access. Open esophagectomy entails a large incisions in the neck, the chest or the abdomen (when all of those areas require an incision it is called a three-field esophagectomy). Minimally invasive esophagectomy is performed through several tiny incisions in the abdomen (laparoscopic) or the chest (thoracoscopic). Esophagectomy may be performed with a stomach-sparing technique, without affecting so much the biliopancreatic area.
	Distal		Known as Ivor-Lewis esophagectomy, it consists of a resection of the distal third of the esophagus including part of the stomach, if involved in disease. Usually this procedure does not give any disadvantage for future endoscopic procedures.
Gastrectomy	Total		Complete removal of stomach with different reconstruction techniques, as creating a pouch, which appears to have better functional outcomes and improved quality of life compared with other types of reconstruction. An Hunt-Lawrence type pouch with a Roux-en-Y jejuno-jejunal anastomosis is the preferred by many surgeons, because it seems to be the most effective. Straight esophagojejunal anastomosis, looped esophagojejunal anastomosis, jejunal interposition, colon interposition, and jejunal pouch construction are different options for restoration of gastrointestinal continuity. Jenjunal pouch reconstruction, can be sited behind (Hunt) or in front (Rodino) of the colon.
	Sleeve		It is a vertically oriented gastrectomy with removal of approximately 70 to 80 percent of the greater curvature of the stomach, resulting in the creation of a narrow gastric tube (the remnant stomach, called sometimes only "sleeve") . It does not affect too much endoscopic procedures.
	Partial	Billroth I	A surgical reconstruction which preserves duodenal and jejunal continuity by an end-to-end anastomosis betwenn the remnant stomach and the duodenal stump.
		Billroth II	An end-to-side anastomosis between the remnant stomach and the proximal jejunum. This reconstruction preserves jejunal but not duodenal continuity, creating a short afferent and an efferent limb.
		Roux-en-Y	Roux-en-Y reconstruction consists of an afferent limb anastomosed to jejunal efferent limb, which is anastomosed with the remnant stomach. It permits a bilious drainage away from the gastric remnant, in order to reduce gastric biliary reflux.
Pancreaticoduodenectomy	Conventional		Conventional pancreaticoduodenectomy (cPD) (i.e. Whipple) consists of a distal gastrectomy with removal of the pancreatic head, duodenum, first 15 cm of the jejunum, common bile duct, and gallbladder. The variant pylorus-preserving pancreaticoduodenectomy keeps the gastric antrum, pylorus, and the proximal 2 to 3 cm of the duodenum, which is anastomosed to the jejunum to restore gastrointestinal continuity.
	Piloro-preserving		Pylorus-preserving pancreaticoduodenectomy (ppPD) preserves the gastric antrum, pylorus, and up to 6 cm of the proximal duodenum, which is anastomosed to the jejunum.
	Subtotal stomach-preserving		The aim of this variant is to preserve as much stomach as possible.
